# Effect of *TP53* deficiency and *KRAS* signaling on the bioenergetics of colon cancer cells in response to different substrates: A single cell study

**DOI:** 10.3389/fcell.2022.893677

**Published:** 2022-09-27

**Authors:** James Kealey, Heiko Düssmann, Irene Llorente-Folch, Natalia Niewidok, Manuela Salvucci, Jochen H. M. Prehn, Beatrice D’Orsi

**Affiliations:** ^1^ Department of Physiology and Medical Physics, Royal College of Surgeons in Ireland, Dublin 2, Ireland; ^2^ RCSI Centre for Systems Medicine, Royal College of Surgeons in Ireland, Dublin 2, Ireland; ^3^ Department of Basic Sciences of Health, Area of Biochemistry and Molecular Biology, Universidad Rey Juan Carlos, Alcorcon-Madrid, Spain; ^4^ Institute of Neuroscience, Italian National Research Council, Pisa, Italy

**Keywords:** metabolic stress, bioenergetics, Cancer Metabolism, colorectal cancer, OxPhos

## Abstract

Metabolic reprogramming is a hallmark of cancer. Somatic mutations in genes involved in oncogenic signaling pathways, including *KRAS* and *TP53*, rewire the metabolic machinery in cancer cells. We here set out to determine, at the single cell level, metabolic signatures in human colon cancer cells engineered to express combinations of activating *KRAS* gene mutations and *TP53* gene deletions. Specifically, we explored how somatic mutations in these genes and substrate availability (lactate, glucose, substrate deprivation) from the extracellular microenvironment affect bioenergetic parameters, including cellular ATP, NADH and mitochondrial membrane potential dynamics. Employing cytosolic and mitochondrial FRET-based ATP probes, fluorescent NADH sensors, and the membrane-permeant cationic fluorescent probe TMRM in HCT-116 cells as a model system, we observed that *TP53* deletion and *KRAS* mutations drive a shift in metabolic signatures enabling lactate to become an efficient metabolite to replenish both ATP and NADH following nutrient deprivation. Intriguingly, cytosolic, mitochondrial and overall cellular ATP measurements revealed that, in WT KRAS cells, *TP53* deficiency leads to an enhanced ATP production in the presence of extracellular lactate and glucose, and to the greatest increase in ATP following a starvation period. On the other hand, oncogenic *KRAS* in *TP53*-deficient cells reversed the alterations in cellular ATP levels. Moreover, cell population measurements of mitochondrial and glycolytic metabolism using a Seahorse analyzer demonstrated that WT KRAS *TP53*-silenced cells display an increase of the basal respiration and tightly-coupled mitochondria, in the presence of glucose as substrate, compared to *TP53* competent cells. Furthermore, cells possessing oncogenic *KRAS*, independently of *TP53* status, showed less pronounced mitochondrial membrane potential changes in response to metabolic nutrients. Furthermore, analysis of cytosolic and mitochondrial NADH levels revealed that the simultaneous presence of *TP53* deletion and oncogenic *KRAS* showed the most pronounced alteration in cytosolic and mitochondrial NADH during metabolic stress. In conclusion, our findings demonstrate how activating *KRAS* mutation and loss of *TP53* remodel cancer metabolism and lead to alterations in bioenergetics under metabolic stress conditions by modulating cellular ATP production, NADH oxidation, mitochondrial respiration and function.

## Introduction

Cancer cells have an altered metabolism which promotes cell survival and proliferation (De Berardinis and Chandel, 2016; Pavlova, Zhu and Thompson, 2022). Such alteration is due to an increased glucose uptake, an exacerbated production of lactate and an inhibition of oxidative phosphorylation system (OXPHOS) that occurs even in the presence of oxygen, thus leading to a state of aerobic glycolysis (Heiden, Cantley and Thompson, 2009). Indeed, tumor cells reprogram their glucose metabolism, restricting it mainly to glycolysis, a phenomenon named as the “Warburg effect” (Geschickter and Warburg, 1930; Warburg, 1956; DeBerardinis and Chandel, 2020). This complex metabolic rewiring is controlled by mitochondria that finely integrate a variety of intracellular signaling pathways, within the mitochondria or with other cellular compartments, to meet bioenergetics needs and facilitate the uncontrolled proliferation of tumor cells (Ward and Thompson, 2012; Epstein, Gatenby and Brown, 2017; Pavlova, Zhu and Thompson, 2022). Glycolytic fueling has been shown to be associated with activating mutations or copy number alterations in genes, such as *RAS, MYC*, or inactivation or repression of genes, such as the tumor suppressor *TP53* (DeBerardinis et al., 2008; Jones and Thompson, 2009). Besides metabolic alterations, these mutations orchestrate several other hallmarks of cancer, including deregulation of cell cycle and cell death pathways, immunosuppression and enhanced migration (Cannino et al., 2018).

Colorectal cancer (CRC) has one of the highest morbidities and mortality rates among solid cancers, accounting for almost 10% of the global incidence of cancer (Sung et al., 2021). CRC is highly heterogeneous at the molecular level and patients display the presence of distinct metabolic alterations that require effective molecular subtyping strategies for therapeutic intervention. Up to 40% of CRC cases contain *RAS* mutations (Lowy and Willumsen, 1993; Andreyev et al., 2001; Scott et al., 2020). The majority of these mutations are *KRAS*, highlighting the importance of studying these defects (Downward, 1998; Matallanas et al., 2011; Slattery et al., 2018). Similarly, dysregulation in *TP53* gene is one of the most frequent events that occurs in approximately 60% of CRC patients and *TP53* mutational status is highly associated with CRC progression and poorer clinical outcome (Muzny et al., 2012; Robles, Jen and Harris, 2016; Nakayama and Oshima, 2019).

Despite researchers’ efforts in understanding the impact of somatic gene mutations in *KRAS* and *TP53* on bioenergetics, cell proliferation and survival in CRC, the dependence of these mechanisms on the mutational status of the tumor still needs elucidation. In the present study, we investigate, at the single cell level, metabolic signatures in isogenic pairs of human colon cancer HCT-116 cells, which differ by single or double genetic mutations in *KRAS* and *TP53* genes. Employing single cell time-lapse imaging approaches, we here show, how *KRAS* and *TP53* mutations influence bioenergetic parameters, including cellular ATP, NADH and mitochondrial membrane potential dynamics following extracellular nutrients availability.

## Materials and methods

### Material

Lipofectamine 2000 and Tetramethylrhodamine methyl ester (TMRM) were obtained from Bio Sciences. All other chemicals, including RPMI 1640 medium, Fetal Bovine Serum, d-glucose, lactate and Carbonyl cyanide 4-(trifluoromethoxy)phenylhydrazone (FCCP) were purchased from Sigma Aldrich.

### Cell lines

All HCT-116 human colon cancer cells were maintained in RPMI 1640 medium supplemented with 10% fetal bovine serum, 100 μg/ml penicillin/streptomycin, 2 mM glutamine, and cultured at 37°C in a humidified atmosphere of 5% CO_2_. Several clones of HCT-116 have been employed and are listed in Table 1. In detail, HCT-116 WT cells were isolated from a patient with colorectal carcinoma and a mutation in codon 13 of the *RAS* proto-oncogene (*KRAS* ^*G13D*^) of these tumor cells is present. *TP53*-deficient HCT-116 (p53 KO) cells were kindly provided by Prof. B. Vogelstein (The Johns Hopkins University School of Medicine, Baltimore, MD, United States) (Bunz et al., 1998; Sur et al., 2009). Hke3 cells were generated from HCT-116 by a somatic deletion of the *KRAS* ^*G13D*^ allele, reverting the oncogenic KRAS phenotype (Shirasawa et al., 1993; Charitou et al., 2019). However, a recent study has shed further light on the accurate *KRAS* status revealing that the Hke3 cell line is *KRAS* dosage mutant, with expression and activity of the *KRAS* mutation approximately 70% lower when compared to the parental cell line (Fasterius et al., 2017). Hke3 cells were kindly provided by Prof. W. Kolch (Conway Institute of Biomolecular and Biomedical Research, University College Dublin, Dublin, Republic of Ireland) (Fasterius et al., 2017; Charitou et al., 2019). p53 has been knocked down in-house by a commercial lentiviral shRNA targeting *TP53* (TRCN0000003753, Sigma) in Hke3 cell lines. These cells were subsequently selected by applying puromycin (15 μg/ml) to generate stable cell lines (Hke3 p53 KD). These pairs of cell lines are considered isogenic and constitute an ideal setting to study phenotypic heterogeneity derived from single mutations. For the purposes of comparison, the Hke3 and Hke3 p53 KD cells are referred to as KRAS WT in this manuscript, as seen in Table 1.

**TABLE 1:** Origin and mutations of human colorectal cancer HCT-116 cell lines.

Cell line	Mutational status	Origin	Disease
HCT-116 WT	*TP53* WT	Primary tumor	Colorectal carcinoma
	*KRAS* ^*G13D*^ mutation		
HCT-116 p53 KO	*TP53* KO	Generated from HCT-116 WT disrupting the two *TP53* alleles	
	*KRAS* ^*G13D*^ mutation		
HCT-116 Hke3	*TP53* WT	Generated from HCT-116 WT by a somatic deletion of the *KRAS* ^*G13D*^ allele	
	*KRAS* WT		
HCT-116 Hke3 p53 KD	*TP53* KD	Generated from HCT-116 Hke3 by *TP53* shRNA silencing	
	*KRAS* WT		

### Plasmids and transfections

Prior to experiments, cells were first seeded in sterile 12 mm Willco dishes (Willco Wells B.V.) for 24 h before transfection using Lipofectamine 2000 (Bio Sciences) as per the manufacturer’s instructions. For cytosolic and mitochondrial ATP measurements, cells were transfected with a vector expressing the genetically-encoded FRET-based cytosolic (ATeam; AT1.03/pcDNA3.1) and mitochondrial (ATeam 1.03R122K/R126K) ATP indicators, respectively [kindly supplied by Dr Hiroyuki Noji (Imamura et al., 2009)]. The ATP-sensitive FRET probes consist of variants of CFP (mseCFP) and YFP (cp173-mVenus) connected by the ɛ subunit of Bacillus subtilis FoF1-ATP synthase. Upon ATP level changes, the ɛ subunit retracts the two fluorophores close to each other, which increases FRET efficiency (Imamura et al., 2009). For cytosolic NADH levels, cells were transfected with a pcDNA3.1-Peredox-mCherry Plasmid (#32383, Addgene). The Peredox-mCherry NADH sensor has been developed by fusing a circular permuted, monomeric T-Sapphire (cpmTS) as fluorescence reporter to a T-Rex tandem dimer (Hung et al., 2011). The Peredox sensor was mainly sensitive to the [NADH][NAD^+^] ratio and resistant to pH changes or other metabolites with structural similarity to NADH. When NAD^+^ binds to the Rex subunits there is a minimal fluorescence emission. In contrast, when NADH binds to the subunits, a conformational change in the probe occurs, leading to a large increase in the T-Sapphire fluorescent signal (Hung et al., 2011).

### Time-lapse live cell imaging

#### Experimental treatments

**FIGURE 1:**
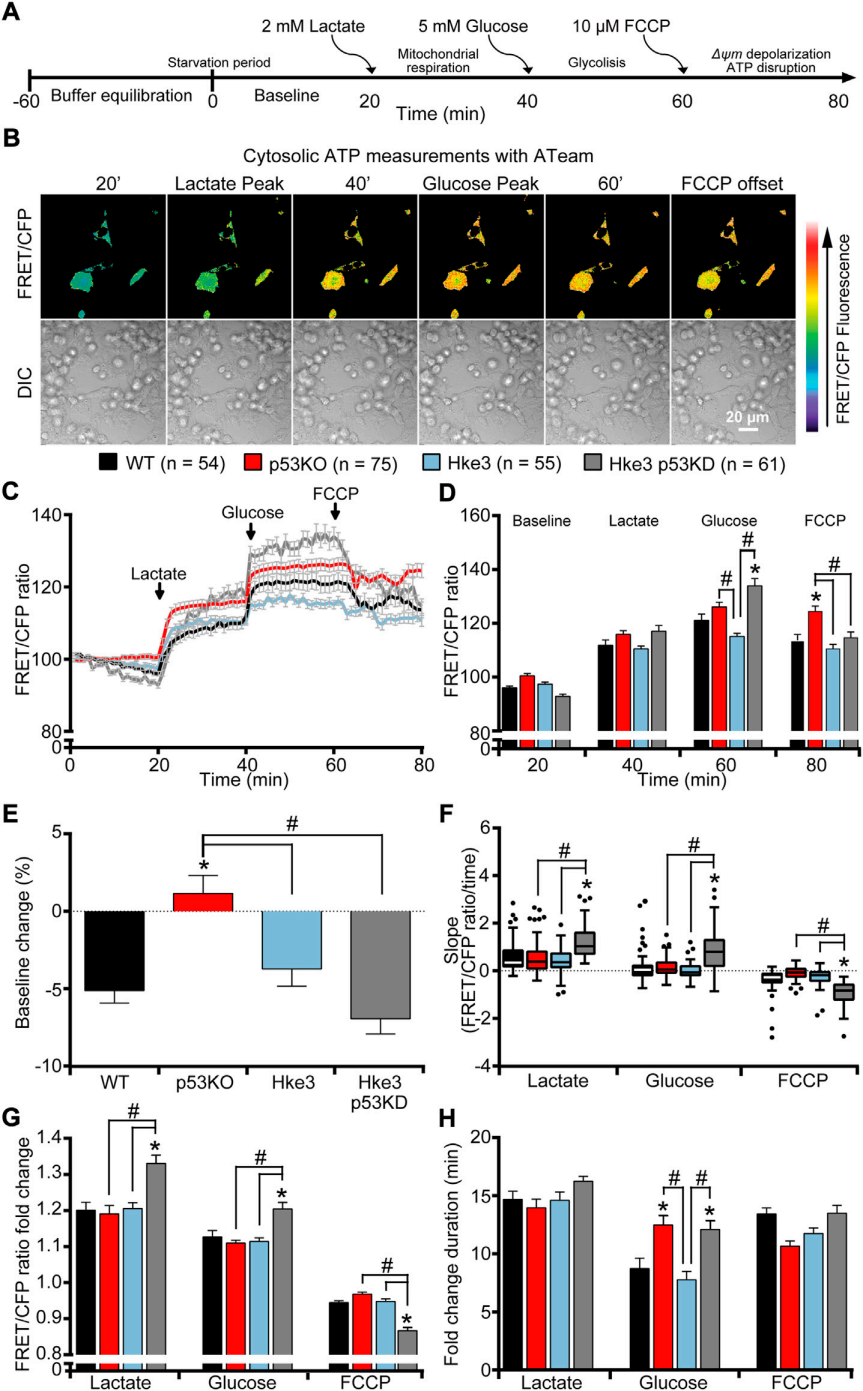
*TP53*-silenced Hke3 cells with a competent *KRAS* gene display significantly increased cytosolic ATP kinetics in response to extracellular substrates addition. WT, p53 KO, Hke3 and Hke3 p53 KD HCT-116 cells were separately transfected with the cytosolic ATP-sensitive (ATeam) FRET probe, loaded with 30 nM TMRM as a Δ*ψ*_m_ indicator (non-quenched mode), mounted on the heated stage of an LSM 710 confocal microscope and assayed over 80 min at 37°C. **(A)** Experimental model schematic. **C**ells were allowed to equilibrate for 60 min on stage (starvation period), after which alterations in Δ*ψ*_m_ and cytosolic ATP were monitored in single cells. Following a baseline of 20 min, cells were exposed to sequential additions of 2 mM lactate, 5 mM glucose and 10 µM FCCP at the indicated time points (20, 40 and 60 min) to promote mitochondrial respiration, glycolysis and, Δ*ψ*_m_ depolarization and mitochondrial ATP disruption, respectively. **(B)** Differential interference contrast (DIC) and fluorescence measurements were recorded for TMRM, FRET, CFP, YFP by time-lapse confocal microscopy. FRET/CFP ratio and DIC representative images of Hke3 cells expressing ATeam probe are shown as a ratio of FRET/CFP normalized to baseline to monitor cytosolic ATP. Scale bar = 20 µm. **(C)** Kinetics of all cells monitored, expressed as means ± SEM are shown, additions are labelled with black arrows on top of the graph. **(D)** Analysis of the cytosolic ATP levels (FRET/CFP, normalized) before each drug treatment (20, 40 and 60 min) and at the end of the experiment (80 min). **(E)** Change of cytosolic ATP baseline levels (FRET/CFP, normalized) over the first 20 min, **(F)** quantification of the slope of cytosolic ATP (the change in FRET/CFP ratio over time in minutes), **(G)** the mean fold change in FRET/CFP ratio and **(H)** the mean duration for this change in FRET/CFP ratio after each treatment are illustrated. Means ± SEM are shown from at least three independent experiments for each cell line (WT, n = 54; p53 KO, n = 75; Hke3 n = 55; Hke3 p53 KD, n = 61). *p ≤ 0.05 compared to WT control cells; #*p* ≤ 0.05 between cell lines (ANOVA, *post-hoc* Tukey).

Experiments were carried out as follows and illustrated in Figure 1A: 1) 1 hour before experiment, the medium was removed and replaced with Krebs-HEPES buffer (KB, 140 mM NaCl, 5.9 mM KCl, 1.2 mM MgCl_2_, 15 mM HEPES) containing 30 nM TMRM and 2.5 mM CaCl_2_, during which cells were starved of nutrients and TMRM equilibrates across plasma and mitochondrial membranes; 2) a baseline was recorded for 20 min as equilibration time; 2 mM lactate was added to the KB at the 20 min time-point, which converts to pyruvate promoting mitochondrial respiration; 3) 5 mM glucose was added on stage at the 40 min time-point, which is imported into the cell promoting glycolysis; and finally 4) 10 µM FCCP was added at the 60 min time-point to induce mitochondrial membrane depolarization and cause disruption of ATP synthesis.

### Single-cell cytosolic and mitochondrial ATP measurements with ATeam and mitoATeam, respectively

HCT-116 cell lines, transfected with the FRET-based cytosolic or mitochondrial ATP indicators, ATeam or mitoATeam respectively, were washed twice with KB and the medium replaced with 1 ml of KB containing the membrane-permeant cationic fluorescent probe TMRM (30 nM) and 2.5 mM CaCl_2_. A thin layer of mineral oil was added on top of the KB to prevent evaporation and Willco dishes were placed on the stage of a LSM 710 confocal microscope equipped with a 40 × 1.3 NA (ATeam) or 63 × 1.4 NA (mitoATeam) oil-immersion objective and thermostatically regulated chamber set at 37 °C (Carl Zeiss). TMRM was excited at 561 nm, and the emission was collected in the range of 575–735 nm. CFP was excited at 405 nm, and emission was collected at 445–505 (CFP) and 505–555 nm (FRET). Yellow fluorescent protein (YFP) was excited directly using the 488 nm line of the Argon Laser and detected in the same range used for FRET. Images were captured every 1 min throughout these experiments.

### Cytosolic NADH measurements with the Peredox-mCherry NADH sensor

HCT-116 cells were prepared as above-described and the medium replaced with 1 ml of KB with 2.5 mM CaCl_2_. A thin layer of mineral oil was added on top of the KB to prevent evaporation and the Willco dishes were placed on the stage of a LSM 710 confocal microscope equipped with a 40 × 1.3NA oil-immersion objective and thermostatically regulated chamber set at 37°C (Carl Zeiss). Peredox and mCherry were excited at 405 and 561 nm, respectively, and the emission was collected at 484–572 (T-Sapphire) and 581–727 nm (mCherry). TMRM was not used in the Peredox experiments as the red fluorescence of the TMRM interferes with the mCherry fluorescence of the probe. The binding of NADH does not cause any change in the intensity of the mCherry fluorescence. Thus, the mCherry signal was used to normalize the T-Sapphire signal and the green-to-red fluorescence increases in conjunction with an increase in the [NADH][NAD^+^] ratio (Hung et al., 2011). Images were captured every 1 min throughout these experiments.

### Mitochondrial NADH measurements using NADH auto-fluorescence

Cells, in 1 ml of KB containing TMRM (30 nM) and 2.5 mM CaCl_2_, were transferred to a heated stage above a 40x/1.3 NA Plan-Neofluar of an inverted epifluorescence microscope (Axiovert 200M, Zeiss) controlled by MetaMorph version 6.5 (Universal Imaging Co.). Ultraviolet excitation at 340 nm can be used to investigate NADH levels in the mitochondria (Patterson et al., 2000; Shuttleworth, Brennan and Connor, 2003; Kasischke et al., 2004). The pyridine ring present in NADH absorbs the UV light and naturally emits light at a higher wavelength in a process known as auto-fluorescence. However, this signal is much stronger in the mitochondria than in the cytosol. Experiments were carried out using a 100 W Mercury short-arc lamp (HBO 103 W/2, Osram, Germany) for excitation with illumination wavelength of 340 nm for NADH excitation with an exposure time of 500 ms and a ND filter of 6% in the excitation light path. Emission was collected at 450–480 nm. TMRM was excited at 545 nm, and the emission was collected in the range of 575–735 nm. Images were captured using an EMCCD camera with binning two for NADH auto-fluorescence and full resolution for TMRM and PHC (Ixon EM DU-897-BI, Andor, NI) every 1 min throughout these experiments.

### Imaging analysis

#### General analysis

All microscope settings including laser intensity and scan time were kept constant for the whole set of experiments. Control experiments were carried out and showed that photo toxicity had a negligible impact. All images were processed and analyzed using ImageJ (National Institutes of Health, Bethesda, MD, United States) and MetaMorph Software version 7.5 or version 6.5 (Universal Imaging Co.). Time-lapse sequences were imported into ImageJ (National Institutes of Health, Bethesda, MD, United States) and background was first subtracted from each image. After creating combined images of the three fields of view for each channel sequence, a median filter with a radius of one pixel was applied. The combined images were then processed using MetaMorph Software version 7.5. Single-cell kinetic measurements (time-stamp, fluorescence intensity or ratio) were input to Excel 2010 macros that automatically generated trace characteristics, including fluorescence intensity, area under the curve, percentage difference from baseline, and maxima and minima values after drugs addition. Normalized values were then calculated as a percentage of baseline values. The baseline was calculated as the mean value of the first 10 images in each experiment). Mean ± SEM values are shown in the all kinetic curves and bar charts. Baseline values for each cell line were calculated as the percentage difference between the normalized values at min one and min 20 for each cell (Baseline change (%) = ΔBaseline_t20-t1_*100). When the % baseline fluorescence is decreasing it is displayed as a negative value. Onset values were calculated as the mean fluorescence intensity for all cells in the minute before additions of lactate, glucose, FCCP or at the end of the experiment, *i.e.* at 20, 40, 60 and 80 min [Onset = (FRET/CFP)_tx_]. Slopes were analyzed by calculating the slope of the kinetic trace for each cell in the corresponding time frame as change in FRET/CFP ratio over time in minutes [Slope = *Δ*(FRET/CFP)/ΔTime with FRET/CFP ratio value for the corresponding time period (21st and 40th min for lactate, 41st and 60th min for glucose, 61st and 80th min for FCCP]. Independent of the curve shape an overall drop of the signal within this time frame will result in a negative slope, while an overall increase will result in a positive slope. In cases where kinetic responses to nutrients or FCCP were close to a linear curve the slope reflects the overall kinetics. We consider that this is the case for the decrease in ATeam and mitoATeam after FCCP, and the increase for mitoAteam after lactate addition, as well as the increase for TMRM after lactate and glucose addition. Data were then grouped per exposure period (baseline, lactate, glucose and FCCP) and displayed as a Tukey box plot of all cells for each cell line. In a Tukey box plot the center line displays the median value with the whiskers showing the lowest datum, within the 1.5 interquartile range of the lower quartile and the highest datum, within the 1.5 interquartile range of the upper quartile. Any data points outside of this range are displayed as a single point. Fold changes were calculated by comparing the peak intensity of each cell post treatment to the intensity in the minute prior to the treatment [Fold Change = Max (FRET/CFP)_Δt_/(FRET/CFP)_tx_]. In the case of FCCP causing a decrease in fluorescence signal, the endpoint was taken as the point where the decrease was no longer exponential in nature (FCCP Offset = Plateau FRET/CFP_Δt_). Change durations were obtained by noting the time taken for each cell to reach its peak/lowest intensity after each addition (Change Duration = Fold Change Δt). All experiments were performed a minimum of three times from independent cell cultures. To analyze single-cell behavior, individual response was treated as an independent event in statistical analyses.

### ATeam, mitoATeam, Peredox-mCherry NADH probes and auto-fluorescence analysis

For cytosolic NADH and ATP measurements, the data were obtained ratiometrically from the different image channels. Regarding the Peredox mCherry NADH sensor, the mCherry signal was used to create a mask which was multiplied by the T-Sapphire signal. The result was then normalized to the mCherry signal. For the ATeam and mitoATeam analysis, the YFP signal was used to create a mask which was multiplied by the FRET signal. The result was then divided by the CFP signal. A custom made MetaMorph journal was used to obtain the average intensity signal from all regions, and an excel macro was then applied to sort the values and to convert them to a percentage normalized to the baseline. Mitochondria within cells were segmented from background using the YFP time lapse images. The segmented mitochondrial areas were converted into a mask used to remove background values from any further analysis of the FRET/CFP stack. To this end, the FRET image stack was first multiplied by the YFP-mask and divided by CFP image stack, and regions of interest were then selected for analysis. For mitochondrial NADH measurements, the values were taken from the resulting images. All experiments were performed at least three times independently of each other.

### Cellular ATP quantification

Cellular ATP was quantified using CellTiter-Glo^®^ luminescence assay (Promega). Cells were seeded at a density of 25,000 cells in a 96 well plate and left in the incubator to adhere overnight. Following this incubation time, the medium was removed and replaced with 100 μl kB buffer. Four control wells containing buffer were also prepared without cells to obtain a value for background luminescence. ATP was quantified following the manufacturer’s instructions. Briefly, 100 μl of the assay buffer was added to each well (containing 100 μl of medium) and cells were incubated for 2 min at RT on a shaker. The plate was left at RT for another 10 min to stabilize the signal, and the contents transferred to a black bottom 96 well plate and then loaded on a Clariostar reader (BMG Labtech) to measure luminescence with a settling time of 0.2 s and the top optic. Protein concentration was assessed with micro BCA (bicinchoninic acid) assay (Pierce) in order to account for differences in cell number. Serial fold dilutions of ATP were prepared to generate an ATP standard curve and moles of ATP normalized to protein concentration.

### Metabolic profile analysis

Metabolic studies were performed using Seahorse XF96 Extracellular Flux Analyzer (Seahorse Bioscience) (Qian and Van Houten, 2010; Lucantoni et al., 2018) to perform either mitochondrial or glycolytic stress tests, according to the manufacturer’s instructions. Hke3 and Hke3 p53 KD cells were plated in XF96 V7 cell culture at 10×10^4^ cells/well and incubated for 48 h in a 37°C, 5% CO2 incubator in RPMI medium. Cells were equilibrated with Seahorse XF DMEM Medium, pH 7.4 (Agilent) for 1 h immediately before extracellular flux assay. Drugs were prepared in the same medium in which the experiment was conducted and were injected from the reagent ports automatically to the wells at the times indicated. During glycolytic stress tests, inhibitors were injected sequentially as follows at the indicated times: 10 mM glucose (G), 1 μM Antimycin/1 μM Rotenone (A/R) and 20 μM Monensin (M). Extracellular acidification rate (ECAR) was measured in mpH/min normalized to protein concentration (μg) to provide measures of bulk glycolytic capacity, allowing determination of glycolysis. Mitochondrial function in Hke3 and Hke3 p53 KD cells was determined through sequential addition of 3 μM Oligomycin (O), 0.5 μM FCCP (F), and 1 μM Antimycin/1 μM rotenone (A/R). Oxygen consumption rate (OCR) was measured in pmolO_2_/min normalized to protein concentration (μg) to provide bulk measures of oxidative phosphorylation. This allowed the determination of basal oxygen consumption, oxygen consumption linked to ATP synthesis (ATP), non-ATP linked oxygen consumption (proton leak), mitochondrial uncoupled respiration (MUR), and non-mitochondrial oxygen consumption (NM), in this order (Qian and Van Houten, 2010; Mookerjee et al., 2017). Basal respiration was calculated by subtracting non-mitochondrial respiration from OCR after the initial stabilization (third measurement), and was considered 100%.

### High content screening microscopy (HCS)

Cells were seeded in a Nunc Micro Well 96 well optical bottom plate (Thermo Scientific) at a density of 1×10^4^ cells per well. The day of the treatment cells were incubated in medium with 1 μg/ml Hoechst 33,588 and 1 μg/ml Propidium iodide (PI). After 0, 24 and 48 h treatment, plates were imaged at 30 fields of view per well using a Cellomics Arrayscan VTI (Thermo Scientific) microscope set up with a temperature of 37°C and 5% of CO_2_ in humidified atmosphere. Images were taken at a resolution of 0.645 μm/pixel using a ×10 Plan-Apo objective lens (NA 0.45), a LED light source set to 20% output (Lumencor Sola, AHF, Germany) and a monochrome CCD camera (Orca-AG, Hamamatsu Photonics, Hertfordshire, UK). The following filters sets were used: Hoechst excitation 387 ± 11 nm, emission 447 ± 30 nm; PI excitation 560 ± 12 nm, emission 620 ± 60 nm all using a HC-Quad band beam splitter with transition wavelength of 410, 504, 582, and 669 nm (Semrock, AHF, Germany). Images were analyzed using a customized processing pipeline to identify nuclei with Hoechst staining (total cell number) and nuclei of dead cells (PI positive) using CellProfiler r2.2.0 (Carpenter et al., 2006). Cellular viability was assessed at basal conditions or 24 and 48 h following 5 mM glucose or high-glucose concentration (11 mM).

### Statistical analysis

Data are given as means ± S.E.M (standard errors of the means). Data were analyzed using one-way analysis of variance (ANOVA) followed by Tukey’s *post hoc* test or Student’s t-test for two-group comparison. *p* values <0.05 were considered to be statistically significant. When significant, exact *p* values were stated in the figure legends.

## Results

*TP53* deficiency enhances cellular ATP production during metabolic stress and the concomitance of oncogenic *KRAS* reverses this alteration in colon cancer cells.

In order to investigate how human colon cancer HCT-116 cells, that harbor mutations in *KRAS* and *TP53* genes, respond to nutrients availability from the extracellular microenvironment, we started our experiments by characterizing parental HCT-116 WT (*TP53* competent but holding a *KRAS* mutation on exon two codon G13) and three isogenic derived mutant cell lines covering all four combinations of *TP53* and *KRAS* mutational status (p53 KO, *TP53*-deficient and *KRAS* mutated; Hke3, *TP53* and *KRAS* competent and Hke3 p53 KD, *TP53-*silenced and *KRAS* competent) as listed in Table 1 [see also Supplementary Figure S1A and materials and methods (Brattain et al., 1984; Shirasawa et al., 1993; Bunz et al., 1998; Sur et al., 2009; Fasterius et al., 2017; Charitou et al., 2019)]. To assess whether *TP53* loss and mutated *KRAS* may affect intracellular and mitochondrial bioenergetics in this colon cancer cell model, we monitored cytosolic and mitochondrial ATP levels at single cell resolution, using ATP-sensitive FRET probes targeted to the cytosol (ATeam) or mitochondria (mitoATeam), respectively (Imamura et al., 2009). Experiments were conducted in all four HCT-116 cell lines by time-lapse confocal microscopy and cells were exposed to extracellular substrates as shown in Figure 1A. Briefly, to stimulate the consumption of intracellular metabolites for survival, HCT-116 cells were first deprived of nutrients for 60 min, after which a baseline was recorded for 20 min as equilibration recording time (still in nutrients deprivation condition). To promote mitochondrial respiration, glycolysis, mitochondrial membrane potential depolarization and disruption of ATP synthesis in this order, cells were exposed to sequential addition of 2 mM lactate (at the 20 min time-point), 5 mM glucose (at 40 min) and 10 µM OXPHOS uncoupler FCCP (at 60 min), and recorded till 80 min (Figure 1A). The addition of FCCP indicates the loss of mitochondrial contribution to cytosolic ATP.

Individual single cell analysis revealed divergent cytosolic ATP concentration across all cell lines during substrates exposure (Figures 1B–D). We first observed that *TP53*-deficient and *KRAS* mutated (p53 KO) cells retained the cytosolic ATP depletion observed in the first 20 min of baseline, when cells are starved of nutrients, compared to other mutant cells, indicating that these were consuming reserves of ATP present in the cytosol during that period (Figures 1C,E). Moreover, *TP53* and *KRAS* mutations drove a shift in metabolic signatures enabling lactate to become an efficient metabolite to replenish cytosolic ATP following stress induced by nutrient starvation (Figures 1B–D,F). Of note, *TP53*-silenced and *KRAS* competent (Hke3 p53 KD) cells displayed the greatest alterations in cytosolic ATP following substrates addition compared to the other cell lines. In detail, lactate and glucose triggered a rapid and constant cytosolic ATP production while a prompt drop of ATP was observed when Hke3 p53 KD cells were subjected to the mitochondrial protonophore FCCP, as shown by quantification of the FRET/CFP ratio (Figure 1D), analysis of the slope of cytosolic signal (Figure 1F) and the corresponding fold change in fluorescence (Figures 1G,H), suggesting that *TP53*-silenced Hke3 cells generate more cytosolic ATP after metabolic stress.

**FIGURE 2:**
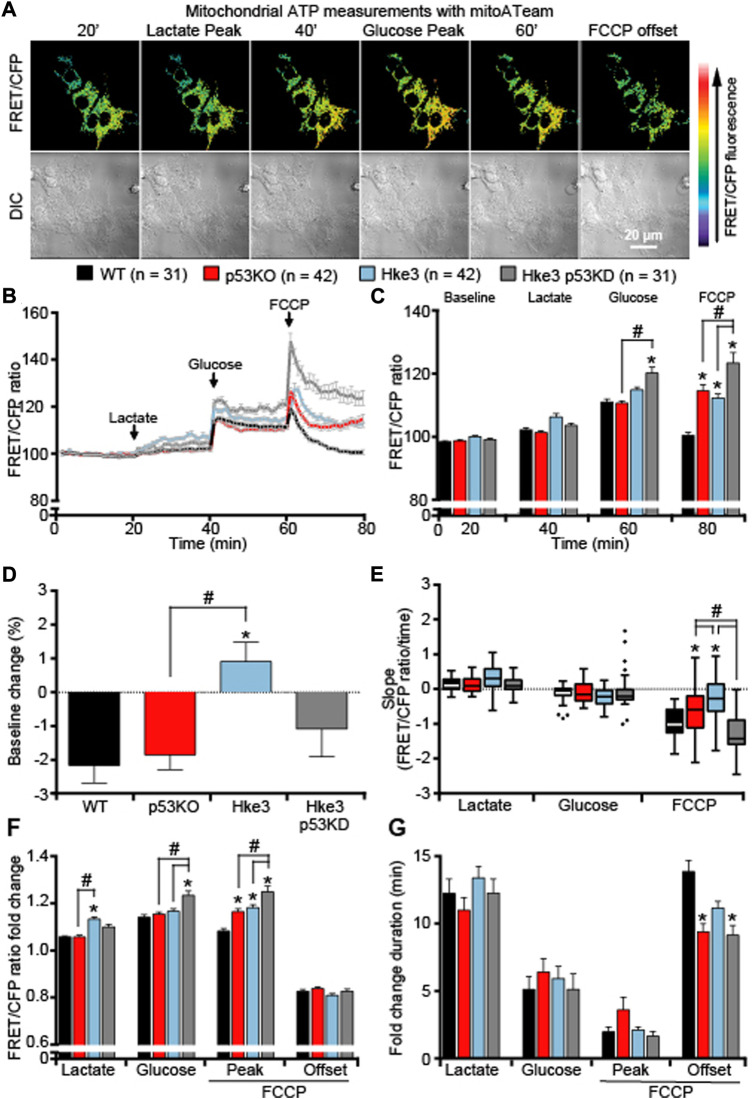
*TP53*-silenced Hke3 cells with a competent *KRAS* show significantly increased mitochondrial ATP levels during metabolic stress. WT, p53 KO, Hke3 and Hke3 p53 KD HCT-116 cells were separately transfected with the mitochondrial ATP-sensitive (mitoATeam) FRET probe, loaded with 30 nM TMRM as a Δ*ψ*_m_ indicator (non-quenched mode), mounted on the heated stage of an LSM 710 confocal microscope and assayed over 80 min at 37°C. **C**ells were exposed as mentioned and illustrated in Figure 1A. **(A)** Differential interference contrast (DIC) and fluorescence measurements were recorded for TMRM, FRET, CFP, YFP by time-lapse confocal microscopy. FRET/CFP ratio and DIC representative images of Hke3 cells expressing mitoATeam probe are shown as a ratio of FRET/CFP normalized to baseline to monitor mitochondrial ATP. Scale bar = 20 µm. **(B)** Kinetics of all cells monitored, expressed as means ± SEM are shown, additions are labelled with black arrows on top of the graph. **(C)** Analysis of the mitochondrial ATP levels (FRET/CFP, normalized) before each drug treatment (20, 40 and 60 min) and at the end of the experiment (80 min). **(D)** Change of mitochondrial ATP baseline levels (FRET/CFP, normalized) over the first 20 min, **(E)** quantification of the slope of mitochondrial ATP (the change in FRET/CFP ratio over time in minutes), **(F)** the mean fold change in FRET/CFP ratio and **(G)** the mean duration for this change in FRET/CFP ratio after each treatment are illustrated. Means ± SEM are shown from at least three independent experiments for each cell line (WT, n = 31; p53 KO, n = 42; Hke3 n = 42; Hke3 p53 KD, n = 31). *p ≤ 0.05 compared to WT control cells; #*p* ≤ 0.05 between cell lines (ANOVA, *post-hoc* Tukey). (H) Total ATP concentration normalized to microgram of proteins for each cell line after 80 min of nutrients starvation. All data are expressed as mean ± SEM from experimental triplicates and three independent cultures. *p ≤ 0.05 compared to WT control cells; #*p* ≤ 0.05 between cell lines (ANOVA, *post-hoc* Tukey).

Similarly, mitochondrial ATP dynamics were determined at single cell level as above-described and analysis of cells monitored showed that, differently to what was detected with the cytosolic ATP-sensitive FRET probe, exposure to lactate to fuel mitochondrial respiration, exhibited minimal changes in mitochondrial ATP in any of the cells regardless of their mutational status (Figures 2A–C,E), indicating that the majority of produced ATP, following lactate addition, was immediately exported into the cytosol. If anything, cells competent in *TP53* and *KRAS* (Hke3) maintained stable mitochondrial ATP basal levels (Figure 2D) and displayed the highest mitochondrial ATP production in response to lactate (Figure 2F). On the other hand, *TP53*-silenced Hke3 and *KRAS* competent cells (Hke3 p53 KD) showed significantly increased mitochondrial ATP levels when glycolysis was promoted (Figures 2B,C,F), and the greatest mitochondrial ATP decrease when mitochondrial ATP synthesis was disrupted, compared to the other mutants and parental cell lines (Figures 2B,C, E-G).

**FIGURE 3:**
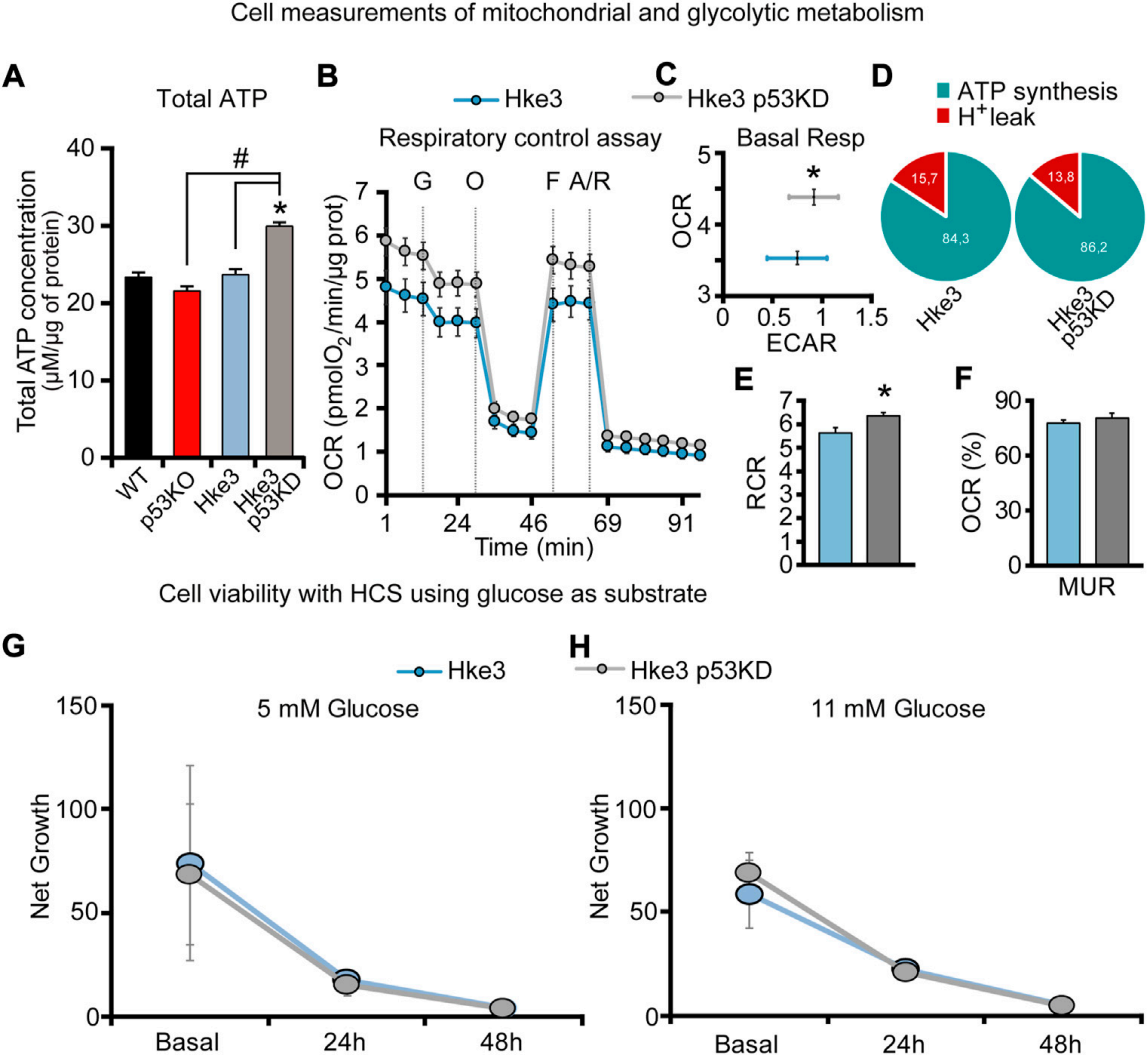
*TP53*-silenced Hke3 cells with a competent *KRAS* display higher basal respiration and tightly-coupled mitochondria. **(A)** Total ATP concentration normalized to micrograms of proteins for each cell line after 80 min of nutrients starvation. All data are expressed as mean ± SEM from experimental triplicates and three independent cultures. *p ≤ 0.05 compared to WT control cells; #*p* ≤ 0.05 between cell lines (ANOVA, *post-hoc* Tukey). **(B–F)** Cellular oxygen consumption rate (OCR) and extracellular acidification rate (ECAR) were measured in Hke3 and Hke3 p53 KD cultured cells in ambient O_2_ concentrations using a Seahorse XF96 Extracellular Flux Analyzer. Sequential injection of substrate (10 mM glucose, G) and metabolic inhibitors Oligomycin (O, 3 μM), FCCP (F, 0.5 μM) and Antimycin/Rotenone (A/R, 1 μM/1 μM) was performed at the indicated times (dashed lines) and enabled determination of bioenergetic parameters. Bioenergetic profiles **(B)** and basal OCR and ECAR **(C)** are represented. ATP synthesis and proton (H^+^) leak parameters were calculated **(D)** and the ratio represented as respiratory control rate (RCR; E). Basal respiration was calculated subtracting non-mitochondrial respiration from OCR after the initial stabilization (third measurement), and it was considered 100%. The percentage of maximal uncoupled respiration (MUR; K) in basal state (non-stimulated conditions) were calculated in relation to basal OCR and determined in the presence of glucose. All data represent mean values ±SEM from four independent experiments from a total of 26–52 wells. OCR and ECAR were normalized to micrograms of protein in each monitored well. *p < 0.05 compared to Hke3 cells (Student’s t test). **(G,H)** Cell net growth (calculated as the ratio of the number of living/proliferating cells (Hoechst 33,588) and dead cells (PI) for Hke3 (in blue) and Hke3 p53 KD (in grey) cultured cells in RPMI with 5 mM (G) and 11 mM (H) glucose. (*p* ≥ 0.05; ANOVA, *post-hoc* Tukey).

In order to confirm whether the ATP production/consumption observed at the single cell microscopy level reflected population-based studies, a biochemical luciferase assay was performed to measure the overall ATP concentration. Indeed, we found that Hke3 p53 KD cells displayed the highest ATP concentration (29.95 μM/μg) following 80 min of starvation at population level compared to the other cells (Figure 3A). In contrast, the lowest overall ATP levels were observed in p53 KO cells (21.58 μM/μg; Figure 3A), indicating that a varying combination of deficiency in *TP53* and mutational *KRAS* status is required to produce significant differences in cellular ATP concentration following nutrient starvation.

Metabolic flux technology using the Seahorse analyzer has emerged to assess the bioenergetic state of cells *in vitro/ex vivo*. For this reason, we also performed glycolytic capacity and respiratory control assays in Hke3 and Hke3 p53 KD cells using a Seahorse analyzer. Cells were sequentially treated as follows at the indicated times (see also Materials and Methods): Glucose 10 mM (G), Oligomycin 3 μM (O), FCCP 0.5 μM (F), and 1 μM Antimycin/1 μM Rotenone (A/R; Figure 3B) and ECAR and OCR were measured. We observed an increase of the basal respiration (Figure 3C) and mitochondrial Respiratory Control Ratio (RCR = ATP synthesis/proton leak; Figures 3D,E), showing tightly-coupled mitochondria, in the presence of glucose as substrate in *TP53*-silenced Hke3 cells compared to *TP53* competent Hke3 cells, implying that loss of *TP53* promoted enhanced mitochondrial function. However, Mitochondrial Uncoupled Respiration (MUR) was not significantly altered (Figure 3F) in Hke3 p53 KD cells compared to their control.

In parallel, to characterize whether *TP53* loss could provide different proliferative properties in Hke3 cells in the absence/presence of challenge, we tested cellular viability using HCS at basal conditions or 24 and 48 h following glucose exposure at normal (5 mM) or high-glucose conditions (11 mM). We observed that *TP53*-silencing in Hke3 cells did not confer a significant proliferative advantage compared to Hke3 cells either in basal conditions or using glucose as a substrate (Figures 3G,H).

Collectively, these experiments demonstrated that *TP53* deficiency enhances ATP production during metabolic stress by nutrients availability and the concomitant presence of oncogenic *KRAS* reverses these ATP alterations (data summarized in Table 2).

**TABLE 2:** Summary of cytosolic, mitochondrial and overall cellular ATP measurements.

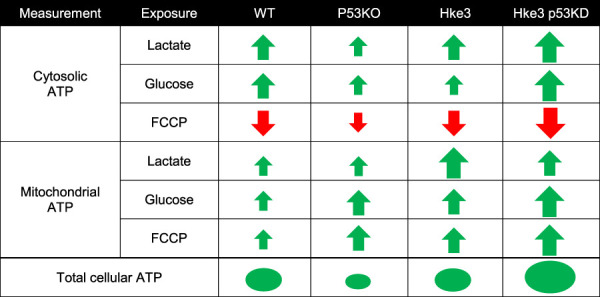

### Oncogenic *KRAS*, independently of *TP53* status, maintains stable mitochondrial membrane potential changes during metabolic stress

**FIGURE 4:**
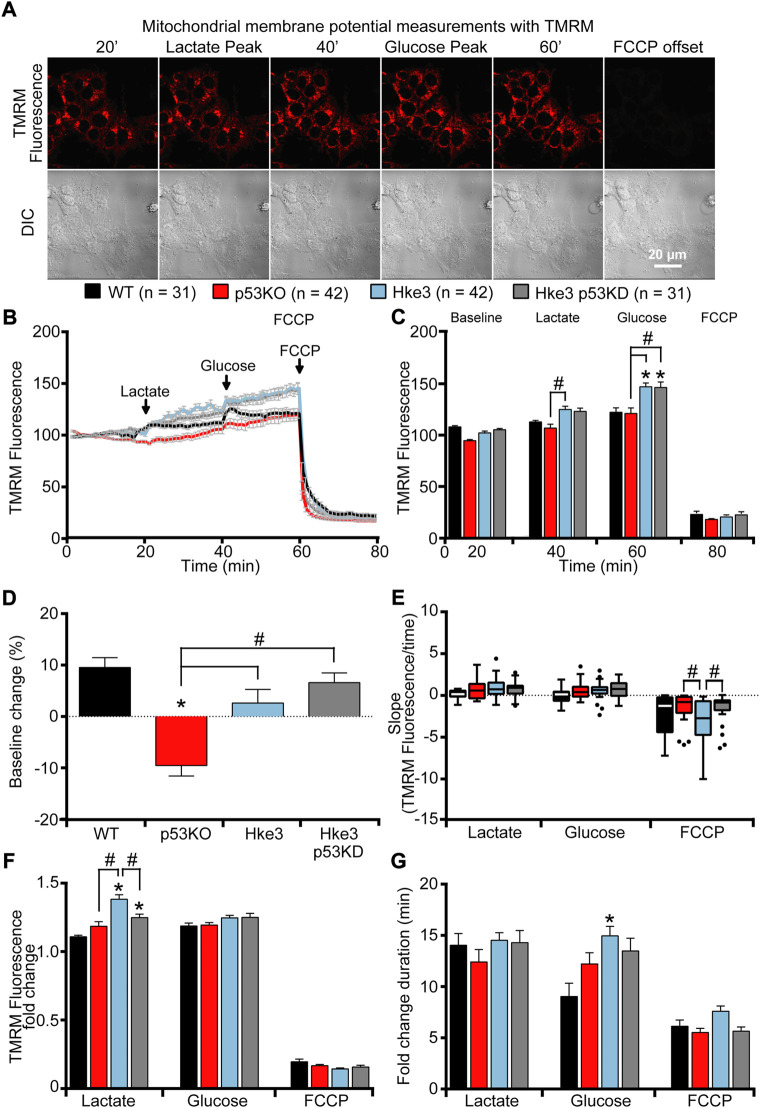
*KRAS* mutation leads to minimal mitochondrial membrane potential changes in response to metabolic substrates. WT, p53 KO, Hke3 and Hke3 p53 KD HCT-116 cells were separately loaded with 30 nM TMRM as a Δ*ψ*_m_ indicator (non-quenched mode), mounted on the heated stage of an LSM 710 confocal microscope and assayed over 80 min at 37°C. Cells were exposed as mentioned and illustrated in Figure 1A. DIC and fluorescent measurements were recorded for TMRM by time-lapse confocal microscopy. Mitochondrial membrane potential imaging data are expressed as a fluorescence intensity normalized to baseline. **(A)** Mitochondrial membrane potential, before and after drugs addition, was analyzed, and TMRM fluorescence and DIC representative images of Hke3 cells are shown. **(B)** Kinetics of all cells monitored are shown as means ± SEM. Scale bar = 20 µm. **(C)** Analysis of TMRM fluorescence levels before each drug treatment (20, 40 and 60 min) and at the end of the experiment (80 min), additions are indicated with black arrows on top. **(D)** TMRM baseline levels over the first 20 min, **(E)** quantification of the slope of TMRM fluorescence (the change in TMRM fluorescence intensity over time in minutes), **(F)** the mean fold change in TMRM fluorescence intensity **(G)** and the mean duration for change in TMRM fluorescence intensity after each treatment are illustrated. Means ± SEM are shown from at least three independent experiments for each cell line (WT, n = 31; p53 KO, n = 42; Hke3 n = 42; Hke3 p53 KD, n = 31). *p ≤ 0.05 compared to WT control cells; #*p* ≤ 0.05 between cell lines (ANOVA, *post-hoc* Tukey).

In parallel with the cytosolic and mitochondrial ATP measurements, we also monitored mitochondrial membrane potential (Δ*ψ*_m_) by employing the membrane-permeant cationic fluorescent probe, TMRM, to determine whether mutations in *KRAS* and *TP53* genes may affect, in human colon cancer cells, also this bioenergetic parameter in response to nutrients availability. Interestingly, TMRM fluorescence intensity decreased only in *TP53*-deficient and *KRAS* mutated (p53 KO) cells over the opening 20 min of the experiment following nutrients starvation (Figures 4B,D), while exposure to lactate and glucose caused a slight rise in TMRM fluorescence in all 4 cell lines (Figures 4A–C). However, cells possessing *KRAS* mutation (WT and p53 KO) showed significantly slower Δ*ψ*_m_ kinetics compared to cells harboring WT *KRAS* (Hke3 and Hke3 p53 KD; Figures 4B,C,F,G), possibly indicative of decreased mitochondrial respiration efficiency. Moreover, a complete Δ*ψ*_m_ depolarization was detected in all 4 cell lines in response to FCCP (Figures 4A–C,E–G), although this was depleted faster in *TP53*-deficient cells (Figure 4E; data summarized in Table 3).

**TABLE 3:** Summary of mitochondrial membrane potential measurements.

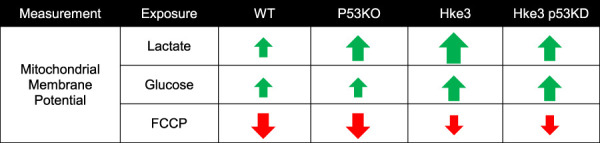

### The double concomitant presence of *TP53* deficiency and oncogenic *KRAS* significantly impairs cellular NADH responses during metabolic stress

**FIGURE 5:**
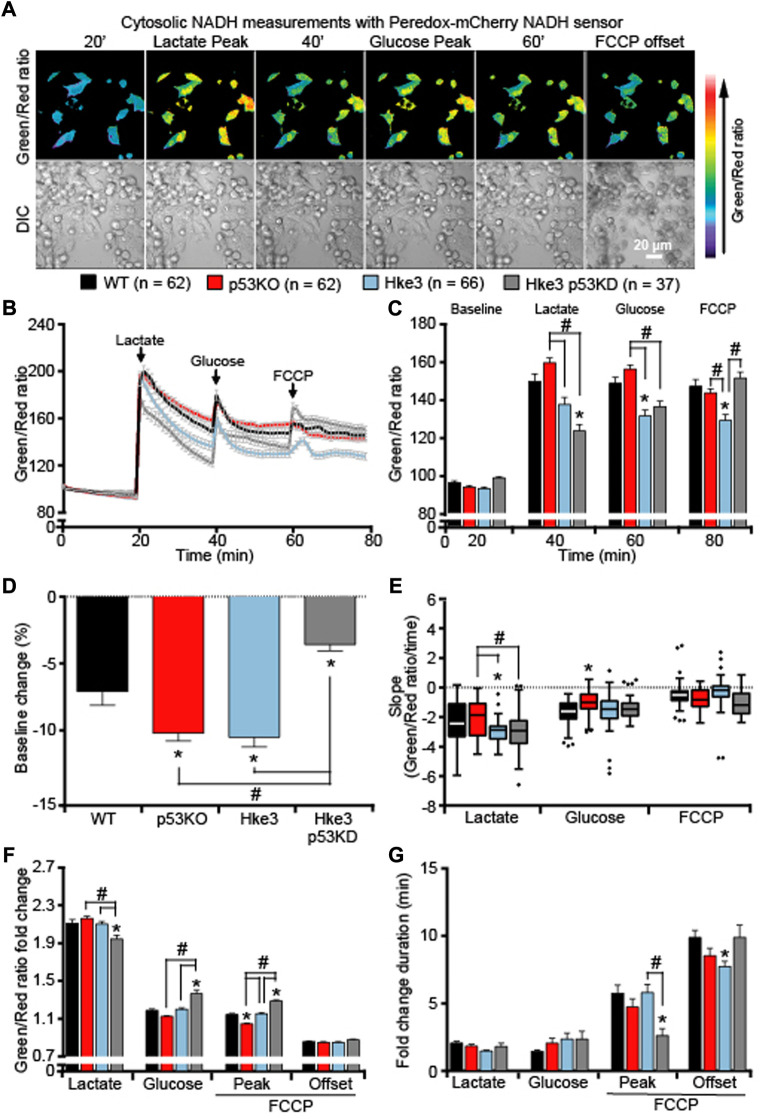
Cytosolic NADH oxidation following substrates addition depends on a correct expression of both *TP53* and *KRAS.* WT, p53 KO, Hke3 and Hke3 p53 KD HCT-116 cells were separately transfected with the cytosolic-targeted NADH-sensitive Peredox-mCherry fluorescent probe, mounted on the heated stage of an LSM 710 confocal microscope and assayed over 80 min at 37°C. Cells were exposed as mentioned and illustrated in Figure 1A. DIC and fluorescent measurements were recorded for GFP and mCherry by time-lapse confocal microscopy. **(A)** Peredox imaging data are shown as a ratio images of Green (excitation with 405 nm)/Red (excitation with 561 nm). Representative DIC and Peredox ratio images of Hke3 cells which report cytosolic NADH are shown before and after drugs addition. Scale bar = 20 µm. **(B)** Kinetics of all cells monitored are shown as means normalized to baseline ±SEM. **(C)** Analysis of the cytosolic NADH levels before each drug treatment (20, 40 and 60 min) and at the end of the experiment (80 min), additions are labelled with black arrows on top of the graph. **(D)** Cytosolic NADH baseline levels over the first 20 min, **(E)** quantification of the slope of cytosolic NADH (the change in Green/Red ratio over time in minutes), **(F)** the mean fold change in Green/Red ratio and **(G)** the mean duration for change in Green/Red ratio after each treatment are illustrated. Means ± SEM are shown from at least three independent experiments for each cell line (WT, n = 62; p53 KO, n = 62; Hke3 n = 66; Hke3 p53 KD, n = 37). *p ≤ 0.05 compared to WT control cells; #*p* ≤ 0.05 between cell lines (ANOVA, *post-hoc* Tukey).

We next analyzed whether the cytosolic and mitochondrial ATP levels observed in colon cancer cells in the presence or absence of somatic mutations in *KRAS* and *TP53* genes were associated with corresponding readings in cytosolic and mitochondrial NADH levels. An identical set of single cell imaging experiments (above-described; Figure 1A) was performed in all four combinations of HCT-116 cells with *TP53* and *KRAS* mutational status following substrates availability. Analysis of cytosolic NADH levels, using a Peredox-mCherry NADH sensor (Hung et al., 2011), revealed heterogeneous cytosolic NADH responses to substrates (Figures 5A–C). In detail, lactate exposure, after a period of starvation, prompted an increase in cytosolic NADH (Figures 5A,B), due to the fact that extracellular lactate is imported into the starved cells and converted into pyruvate to fuel mitochondrial respiration. This was followed by a rapid decrease in the NADH fluorescence ratio across all cell types, suggesting that the malate-aspartate shuttle was functioning to regenerate mitochondrial NADH for ATP production by oxidizing NADH to NAD^+^ in the cytosol. A second increase/decrease in fluorescence ratio was detected immediately after glucose addition (Figures 5A,B); however, this occurred at a different extent compared to lactate exposure as cells were no longer starved and favored glycolysis over mitochondrial respiration. Finally, the addition of FCCP resulted in a third rise/drop in the cytosolic NADH (Figures 5A,B), most likely caused by a reversal of the malate-aspartate NADH shuttle, an event that can occur under stress conditions (Pelley, 2012; Kane, 2014). Quantification of individual cells, at the chosen time-points, revealed that *TP53*-silenced Hke3 and *KRAS* competent cells (Hke3 p53 KD) showed more stable basal cytosolic NADH levels (in the first 20 min) during starvation period (Figure 5D) and significantly decreased NADH levels following lactate exposure (Figures 5B,C,F,G) compared to other mutant cells. Similarly, *TP53* and *KRAS* competent cells (Hke3) displayed reduced cytosolic NADH following glucose and FCCP addition (Figure 5C), highlighting the fact that cells possessing oncogenic *KRAS*, and especially those in which *TP53* is absent, may have an enhanced cytosolic NADH oxidation during metabolic stress. However, cytosolic NADH oxidation occurred more rapidly in Hke3 after lactate addition and more slowly in p53 KO cells (*TP53*-deficient and *KRAS* mutated) after glucose (Figure 5E).

**FIGURE 6:**
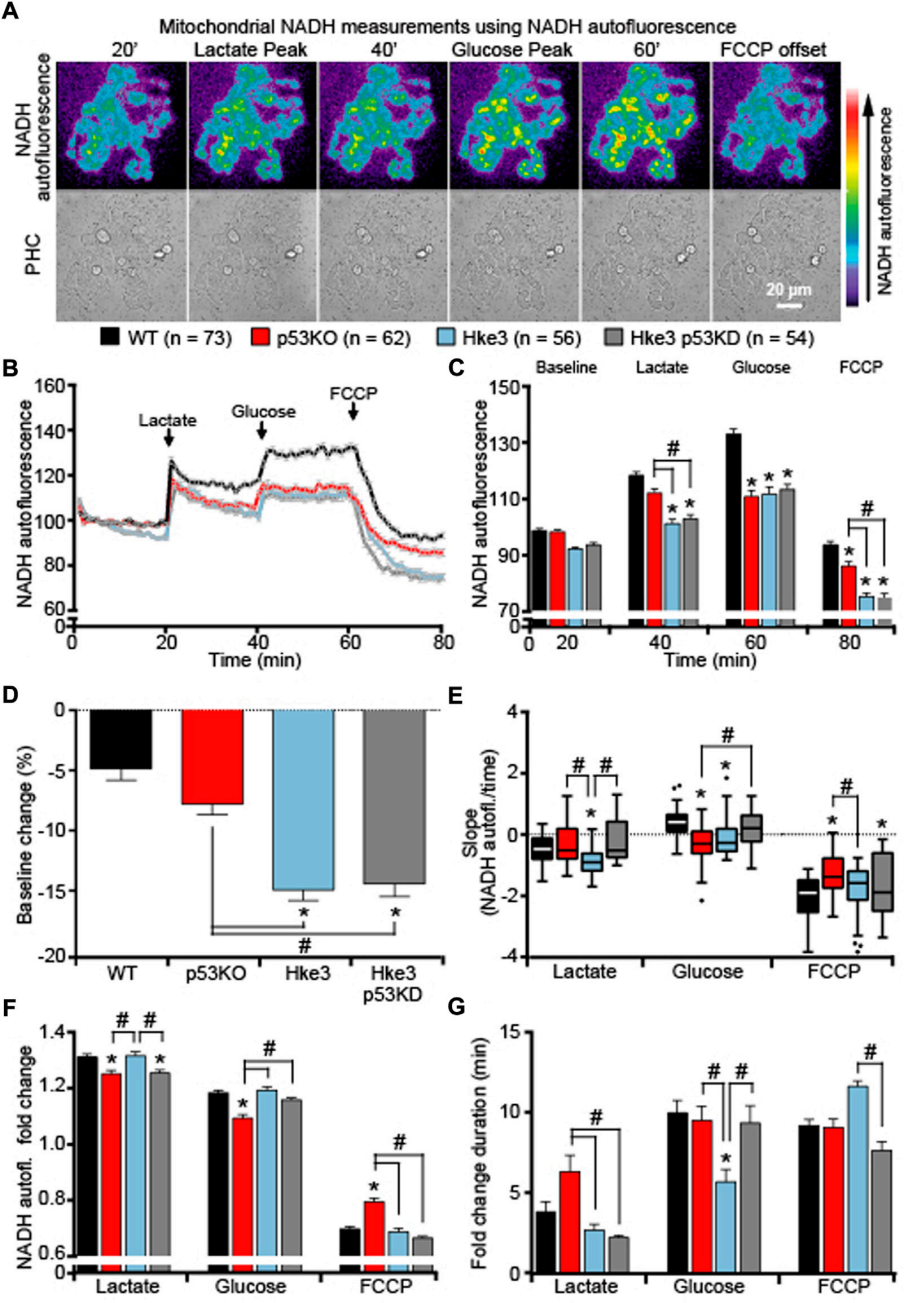
*TP53*-deficient *KRAS* mutated cells display major alterations in mitochondrial NADH in response to metabolic substrates. WT, p53 KO, Hke3 and Hke3 p53 KD HCT-116 cells were separately mounted on the heated stage of an Axiovert 200 M motorized microscope and assayed over 80 min at 37°C. Cells were exposed as mentioned and illustrated in Figure 1A. Fluorescent measurements were recorded for NADH auto-fluorescence and phase contrast (PHC) by time-lapse fluorescence microscope. Fluorescent imaging data are expressed as NADH fluorescence normalized to baseline. **(A)** Representative imaging data of Hke3 cells show the inhomogeneous fluorescence of NADH per cell reflecting the predominant mitochondrial localization and are selected to illustrate levels before and after drugs addition. Scale bar = 20 µm. **(B)** Kinetics normalized to baseline of all cells monitored shown as means ± SEM. **(C)** Analysis of the mitochondrial NADH levels before each drug treatment (20, 40 and 60 min) and at the end of the experiment (80 min), additions are labelled with black arrows on top of the graph. **(D)** Mitochondrial NADH baseline levels over the first 20 min, **(E)** quantification of the slope of mitochondrial NADH (the change in fluorescence intensity over time in minutes), **(F)** the mean fold change in fluorescence intensity and **(G)** the mean duration for change in fluorescence intensity after each treatment are illustrated. Means ± SEM are shown from at least three independent experiments for each cell line (WT, n = 73; p53 KO, n = 62; Hke3 n = 56; Hke3 p53 KD, n = 54). *p ≤ 0.05 compared to WT control cells; #*p* ≤ 0.05 between cell lines (ANOVA, *post-hoc* Tukey).

Having observed how *KRAS* and *TP53* status altered cytosolic NADH responses, we then examined mitochondrial NADH kinetics following metabolic stress and substrates addition. As the oxidized form NAD^+^ does not emit visible fluorescence, it is possible to use bound NADH in a cell as a direct indicator of cellular respiration (Chance, 1954; Chance and Thorell, 1959) and exploit its auto-fluorescence to directly monitor mitochondrial NADH (Duysens and Amesz, 1957; Lucantoni et al., 2021). This is also sensitive to NADPH; however, the main auto-fluorescence signal derives from the NADH inside the mitochondria where, in fact, we find the highest signal. Quantification of NADH auto-fluorescence indicated significant decreased mitochondrial NADH following nutrients deprivation (Figures 6A–D), substrates addition and FCCP (Figures 6A–C) in all cells regardless of their mutational status compared to parental cells (WT) in which only *KRAS* mutation is present. However, these reduced NADH alterations were especially pronounced in cells possessing an active *KRAS*, independently of *TP53* status (Figures 6B–D), suggesting that mitochondrial NADH is enhanced by the presence of oncogenic *KRAS* during metabolic stress. Moreover, analysis of the slope of mitochondrial signal revealed that mitochondrial NADH oxidation occurs faster in cells competent in *TP53* and *KRAS* (Hke3) following lactate, glucose and FCCP addition and slower in *TP53*-deficient cells, regardless *KRAS* status, after lactate and FCCP (Figure 6E). Nevertheless, evaluation of the corresponding fold change in fluorescence showed a reduced mitochondrial NADH response particularly in cells simultaneously harboring deficient *TP53* and mutated *KRAS* (p53 KO; Figure 6F. G).

All together, these data exhibited that oncogenic *KRAS* enhances cytosolic and mitochondrial NADH and the presence of *TP53* coexistent mutational status may trigger the major alterations in cellular NADH levels during metabolic stress (please see Table 4 for a summary of key findings).

**TABLE 4:** Summary of cytosolic and mitochondrial NADH measurements.

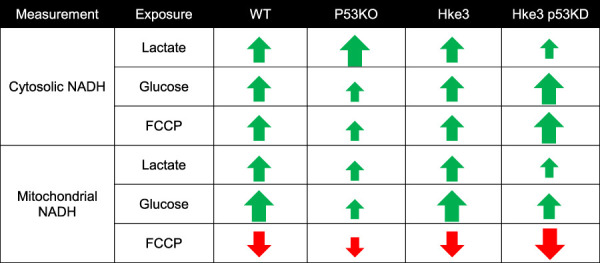

## Discussion

In the present study, we identified how tumor suppressors and/or oncogenic mutations, such as *KRAS* and *TP53* genes, alter cancer metabolism, and specifically how they rewire cellular bioenergetic parameters. Using genetically engineered human colon cancer HCT-116 cells in combination with single cell imaging approaches, we demonstrated that *TP53* and *KRAS* mutations and nutrients availability from the extracellular microenvironment affect cellular ATP, NADH and mitochondrial membrane potential dynamics both at cytosolic and mitochondrial level. First, we showed that, in WT *KRAS* cells, *TP53* deficiency leads to the greatest increase in ATP following a period of nutrient starvation and an enhanced ATP production in the presence of substrates, such as lactate and glucose. Conversely, *KRAS* mutation in *TP53*-deficient cells reversed these alterations. Moreover, *KRAS* mutation, independently of *TP53* status, produces minimal mitochondrial membrane potential variations in response to nutrients availability. Finally, we determine that the simultaneous presence of *TP53* deficiency and *KRAS* mutation triggers significant shifts in cytosolic and mitochondrial NADH levels during metabolic stress induced by nutrients deprivation or accessibility.

*KRAS* and *TP53* somatic mutations are two of the most common and well characterized mutations in CRC, a disease that develops in a stepwise fashion in terms of genetic mutations, activation of oncogenes and loss of function of tumor suppressing genes (Vogelstein et al., 1988; Fearon and Vogelstein, 1990; Smith et al., 2002; Hasbullah and Musa, 2021). In the recent years, several studies have been focused in understanding the effects of *KRAS* mutation and *TP53* loss in numerous cell models and systems, largely demonstrating that they play a role in cancer cell energy metabolism through regulation of processes, such as glucose transport, lactate metabolism and mitochondrial function. In detail, it has been shown that oncogenic *KRAS* alters ATP production, decreases aerobic respiration and increases ROS generation, while *TP53* dampens glycolysis and modulates OXPHOS and glutamine metabolism (Chen and Russo, 2012; Tarrado-Castellarnau, de Atauri and Cascante, 2016). Moreover, other research groups revealed that *KRAS* mutation, on its own, mainly increases cell proliferation, reprograms metabolic pathways and affects mitochondrial metabolism (Weinberg et al., 2010; Gaglio et al., 2011; Pylayeva-Gupta, Grabocka and Bar-Sagi, 2011; Simanshu, Nissley and McCormick, 2017). Similarly, p53 influences metabolic signaling through a number of different mechanisms, including the interaction with substrates and enzymes involved in energy metabolism (Vousden and Prives, 2009; Frezza and Martins, 2012; Berkers et al., 2013; Kruiswijk, Labuschagne and Vousden, 2015), *i.e* the activation of p53 by malate dehydrogenase (MDH) in response to low glucose (Lee et al., 2009) or increasing flux through the pentose phosphate pathway (PPP), which promotes anabolism needed for cell growth (Buzzai et al., 2007). Our study supports these previously reported activities, but also gives new important insights in *KRAS* and *TP53* role in cancer metabolism. In fact, our data obtained from single cell imaging experiments suggested that *TP53* deficiency increases cytosolic, mitochondrial and overall cellular ATP production during metabolic stress induced by nutrients availability, including substrates deprivation, glucose and lactate exposure, in HCT-116 colon cancer cells (Figures 1, 2 and Table 2). It is worth to note that cytosolic ATP depends on the amount of ATP produced and cellular ATP consumption. The kinetics of the change in cytosolic ATP concentration is then an indicator how fast this new equilibrium is reached. However, we have also to consider a change of mitochondria from ATP producer to ATP consumer with the possibility of a reversal of the ATP synthase after mitochondrial membrane potential depolarization. For example, mitochondrial ATP in cells with lactate and glucose as metabolites, treated with FCCP, increased which is an indicator that, at least the uptake of ATP from the cytosol into the mitochondrial matrix took place (a requirement for the ATP synthase reversal). Nevertheless, this did not seem to lead to a recovery of mitochondrial membrane potential which would have been indicated by an increase in TMRM nor did it lead to a recovery of mitochondrial NADH. Moreover, cell population measurements of mitochondrial and glycolytic metabolism in Hke3 and Hke3 p53 KD cells using a Seahorse analyzer demonstrated that *TP53*-silenced Hke3 cells display an increase of the basal respiration and tightly-coupled mitochondria, in the presence of glucose as substrate, compared to *TP53* competent Hke3 cells (Figure 3). It is interesting in this context that CRC cells may rely more on OXPHOS than glycolysis compared with normal colon cells (Kaldma et al., 2014; Chekulayev et al., 2015). However, as all population-based assays, this technology is lacking high-temporal resolution. In addition, measurement of bioenergetics in individual cells, their cell-to-cell heterogeneity, and measurement of bioenergetics in specific cellular compartments (such as cytosol *vs* mitochondria) are only possible at single-cell resolution. Single cell analyses employing cytosolic and mitochondrial FRET-based ATP probes, fluorescent NADH sensors, or probes such as TMRM also enable dynamic computational modelling of cellular bioenergetics in dependence on their mutational status, allowing the identification of new therapeutic targets.

To date, what is known in literature is that glycolytic HCT-116 *TP53*-deficient cells generate more ATP when compared to *TP53*-proficient cells (Matoba et al., 2006) and *TP53* deficiency can increase metabolites flux through PPP activity, thereby reducing substrates for ATP production during glycolysis (Flöter, Kaymak and Schulze, 2017). p53 also responds to changes in energy levels in cells (Okorokov and Milner, 1999) and it inhibits the expression of glucose transporters GLUT1 and GLUT4, thus dampening glycolysis (Schwartzenberg-Bar-Yoseph, Armoni and Karnieli, 2004; Kondoh et al., 2005), also under hypoxic conditions by inducing RRAD (RAS-related associated with diabetes), the Ras-related small GTPase (Zhang et al., 2014). This would further support our results showing that *TP53*-silenced Hke3 cells (Hke3 p53 KD) import glucose faster than *TP53*-proficient cells (Hke3), leading to a larger increase in fluorescence (Figures 1, 2 and Table 2). This effect is accompanied by the ability of p53 to support mitochondrial fatty acid oxidation (FAO) and drive OXPHOS (Lebedeva, Eaton and Shadel, 2009; Liu et al., 2014). Intriguingly, the concomitant presence of oncogenic *KRAS* in *TP53*-deficient cells entirely inverts the alteration of cellular ATP levels in response to substrates in our system (Figures 1, 2 and Table 2). In line with our findings, transformed *KRAS* mouse fibroblasts displayed lower ATP content, reduced OXPHOS ability and more sensitivity to glucose depletion when compared to their immortalized normal counterparts (F. Chiaradonna et al., 2006a; Ferdinando Chiaradonna et al., 2006b). Oncogenic *KRAS* has also been shown to have significant effects on the activity and function of the mitochondria, suppressing mitochondrial complex I activity (Hu et al., 2012), leading to reduced respiration (Baracca et al., 2010), possibly decreasing mitochondrial ATP generation. Otherwise, *KRAS* mutated cells use OXPHOS-independent ROS generation through complex III for growth, and utilize glucose metabolism to fuel the PPP rather than ATP production (Weinberg et al., 2010). It has also been shown that the activity of NF-κB resulted enhanced in *TP53*-deficient primary mouse embryonic fibroblasts and, in these cells, the oncogenic *RAS*-induced cell transformation and acceleration of aerobic glycolysis were blocked in the absence of NF-κB. However, this was restored by GLUT3 expression, indicating that *TP53* loss can facilitate the glycolysis by upregulating the expression of GLUT3 through NF-κB pathway (Kawauchi et al., 2008). Furthermore, it is known that the glycolytic pathway is regulated by p53 through the expression of Hexokinase II (HK2), which controls the production of Glucose-6-Phosphate (G6P) (Mathupala, Heese and Pedersen, 1997; Flöter, Kaymak and Schulze, 2017). Mutations or deletions in *TP53* in cancers result in the upregulation of both glucose transporters and HK2 and glycolysis by expression of glycolytic enzymes, like PGM, and inhibition of Tumor Protein 53-Induced Glycolysis and Apoptosis Regulator (TIGAR) (DeBerardinis, 2008). Cancer-associated mutations in *TP53* have been shown to result in loss of the ability to block G6PD activity, resulting in an increased PPP flux and glycolysis (Jiang et al., 2011). Another target of *TP53* is Glutaminase 2 (GLS2), which is localized in the mitochondria and hydrolyzes glutamine into glutamate, thereby promoting ATP production through the mitochondrial oxidative phosphorylation (Hu et al., 2010; Liu et al., 2019; Yu et al., 2022). In addition, *KRAS* has also been demonstrated to lead to an increase of glycolytic enzyme expression (Ying et al., 2012; Pupo et al., 2019).

Notably, we also demonstrate that *KRAS* mutation leads to minimal mitochondrial membrane potential changes, detected in terms of alterations in TMRM fluorescence levels, in response to metabolic substrates and this event occurs independently of *TP53* status (Figure 4 and Table 3). Indeed, oncogenic *KRAS* inhibits mitochondrial function by inducing Hypoxia Inducing Factors 1α (HIF-1 α), which causes increased expression of pyruvate dehydrogenase kinase 1 (PDK1) that, in turn, prevents the activity of pyruvate dehydrogenase (PDH), decreasing the amount of pyruvate that enters the TCA cycle and suppressing mitochondrial O_2_ consumption (Kim et al., 2006; Chun et al., 2010; Kaplon et al., 2013; Kierans and Taylor, 2021).

Additionally, our results evidence that, in this human colon cancer cell model, co-operation between KRAS and p53 is crucial in explicating a correct cellular NADH oxidation. In fact, the double concomitant deficiency of *TP53* and mutation in *KRAS* produced significantly altered cytosolic and mitochondrial NADH levels in response to substrates, indicating a clear interplay between them during metabolic stress (Figures 5, 6 and Table 4). In depth, while cytosolic NADH oxidation during lactate exposure occurred rapidly in *TP53* and *KRAS* competent cells (Hke3), thus exhibiting a greater capacity for OXPHOS and superior mitochondrial function, and more slowly during glucose addition in *TP53*-deficient and *KRAS* mutated cells (p53 KO; Figure 5), mitochondrial NADH oxidation resulted faster in *TP53* and *KRAS* competent cells (Hke3) during lactate and glucose exposure (Figure 6), implying that both *TP53* and *KRAS* are needed for a proper mitochondrial function. This could indicate higher lactate dehydrogenase (LDH) activity in *KRAS-*mutated cells or a higher rate of NADH oxidation in WT *KRAS* cells. For instance, some reports have shown that LDH expression and activity are increased in *KRAS* mutated lung cancers (McCleland et al., 2013; Xie et al., 2014) and increased GLUT expression in *KRAS* mutated cells that amplified glycolysis rate in pancreatic cancer (Bryant et al., 2014). Of note, a superior mitochondrial function may lead to increased cytosolic NADH oxidation by the malate-aspartate shuttle, responsible for conversion of oxaloacetate to malate in the inner mitochondrial membrane space and aspartate in the mitochondrial matrix (Birsoy et al., 2015; Sullivan et al., 2015). Oncogenic *KRAS* produces increased expression of the malate dehydrogenase (MDH) and aspartate aminotransferase (GOT), thus regulating the malate-aspartate shuttle activity (Lyssiotis et al., 2013; Hanse et al., 2017). As *TP53*-deficient cells displayed lower increases in NADH fluorescence compared to *TP53*-proficient cells (Figures 5, 6), this may also indicate reduced activity of the malate-aspartate shuttle in cells where *TP53* is absent. Several links between *TP53-*related cancer and cell metabolism and MDH1 have been found (Bensaad et al., 2006; Matoba et al., 2006; Kawauchi et al., 2008; Lee et al., 2009).

In our study, the interplay between different *KRAS* and *TP53* mutational status is obvious and leads to an impairment of both cytosolic and mitochondrial bioenergetics. However, it is worth noting that all our experiments have been performed in atmospheric normoxic oxygen conditions (21%) and we here explore only loss of function of *TP53*. Indeed, we are aware that cancer tissue oxygenation, and especially in the core of the tumor, would be a hypoxic environment (1–4%) and some p53 missense mutant proteins demonstrate an ability to gain new functions.

Long ago, interactions between *RAS* and *TP53* have been demonstrated to contribute to transformation of normal cells (Eliyahu et al., 1984; Parada et al., 1984). Mutant *TP53* has been shown to co-operate with oncogenic *KRAS* in the formation of PDAC tumors in mouse models (Bailey et al., 2016) and in pancreatic cancer metastasis (Weissmueller et al., 2014). Similarly, combination of oncogenic *KRAS* and *TP53* loss triggered lung tumorigenesis and reliance on catabolism of branched-chain amino acids (BCAAs) as nutrients (Mayers et al., 2016) and KRAS has been shown to suppress p53 by activating the NRF2-regulated antioxidant defense system in human lung cancer cells (Yang et al., 2020). Studies on CRC patients have found that co-mutations of both *TP53* and *KRAS* were rare, suggesting alternate pathways for them in the development of CRC (Smith et al., 2002). Conversely, other reports detected co-existing mutations in 26 out of 140 patients (Tortola et al., 1999), and more recently 26% of 229 patients presented mutations of both *KRAS* and *TP53* (Chow et al., 2016) and a mild correlation was observed between the expression of the *KRAS* gene and *TP53* immunoexpression in colorectal adenocarcinoma (Rachmawati et al., 2019).

Rewiring of cellular metabolism represents a fundamental trait of most cancer cells. Beyond its role in providing energy substrates and biomolecules for cell proliferation, cellular metabolism has also been found to be tightly associated with cancer cell fate and phenotype, and to contribute to the complex tumor heterogeneity that exists within the tumor mass (Lee, 2015). Differences in cellular bioenergetics influences how tumor cells interact with immune cells and other cells of the surrounding tumor microenvironment (TME) (Fu et al., 2017). It has been shown that the presence of *TP53* and *KRAS* mutations has strong implications for the function of the surrounding TME (Michel et al., 2021; Nenkov et al., 2021; Pereira et al., 2022), immune signaling and escape (Mantovani et al., 2008; Hamarsheh et al., 2020). Both immune and stromal cells have been shown to be compromised by *TP53*- and *KRAS*-mediated tumor signaling, thereby enhancing tumor development and growth. In CRC, a high prevalence of *KRAS* mutations correlates with chronic inflammatory diseases, and *TP53* mutations contribute to immune surveillance, angiogenesis and remodeling of the extracellular matrix (Michel et al., 2021; Nenkov et al., 2021; Pereira et al., 2022).

In conclusion, our results identify how, in colon cancer cells, the oncogenic mutation in *KRAS* and deletion of tumor suppressor *TP53* rewire cancer metabolism and, in particular, how they co-operate in this system, to alter cytosolic and mitochondrial bioenergetics.

## Data Availability

The raw data supporting the conclusions of this article will be made available by the authors, without undue reservation.
