# Supplementary figures and images for: Effect of TP53 deficiency and KRAS signaling on the bioenergetics of colon cancer cells in response to different substrates: A single cell study
Source: Front Cell Dev Biol. 2022 Sep 27;10:893677. doi: 10.3389/fcell.2022.893677 (PMC9550869; doi:10.3389/fcell.2022.893677)

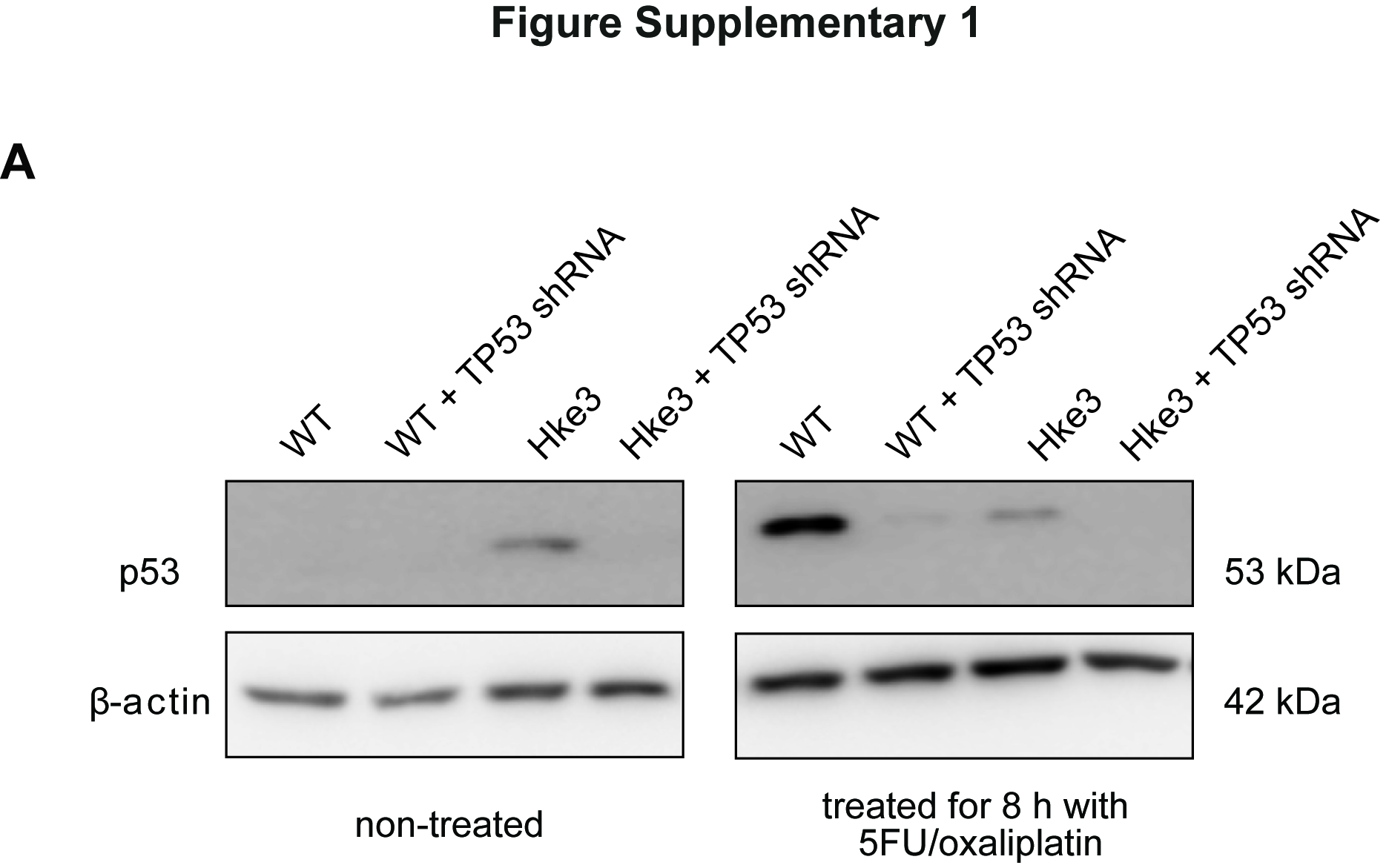

Supplement: Supplementary file 1 [file Image1.TIF]
