# Supplementary material for: Effect of TP53 deficiency and KRAS signaling on the bioenergetics of colon cancer cells in response to different substrates: A single cell study
Source: Front Cell Dev Biol. 2022 Sep 27;10:893677. doi: 10.3389/fcell.2022.893677 (PMC9550869; doi:10.3389/fcell.2022.893677)
Supplement: Supplementary file 2 [file DataSheet1.DOCX]

Supplementary Material

# Supplementary Data

Western blotting

Preparation of cell lysates from HCT-116 WT and Hke3 cells and Western blotting was carried out as previously described ([Reimertz et al., 2001](#_ENREF_58)). The resulting blots were probed with a mouse monoclonal TP53 antibody (DO-1, Novocastra) and a mouse monoclonal β-actin antibody (clone DM 1A; Sigma) diluted 1:5000. Horseradish peroxidase conjugated secondary antibodies diluted 1:10000 (Pierce) were detected using Immobilon Western Chemiluminescent HRP Substrate (Millipore) and imaged using a FujiFilm LAS-3000 imaging system (Fuji).


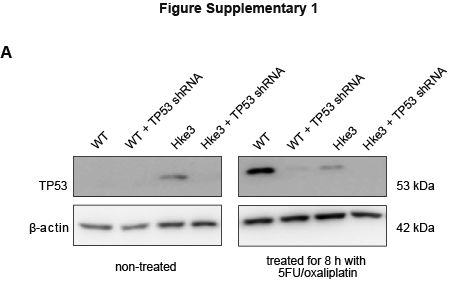


**Figure Supplementary 1. Stable Hke3 p53 KD colon cancer cells.** (A) Western blotting showing TP53 expression in human colon cancer HCT-116 WT and Hke3 cells in the presence and the absence of a lentiviral shRNA targeting *TP53* (TRCN0000003753, Sigma) in non-treated conditions and following 5-FU/oxaliplatin treatment (10 mM of 5-FU/oxaliplatin for 8 hours).

**References**

Reimertz C, Kogel D, Lankiewicz S, Poppe M, Prehn JH (2001) Ca(2+)-induced inhibition of apoptosis in human SH-SY5Y neuroblastoma cells: degradation of apoptotic protease activating factor-1 (APAF-1). Journal of neurochemistry 78:1256-1266.
